# Case report: acute bowel obstruction with an isolated transition point in peritoneal dialysis patients; a presentation of encapsulating peritoneal sclerosis?

**DOI:** 10.1186/s12882-015-0214-2

**Published:** 2016-01-05

**Authors:** Sassan Ghazan-Shahi, Joanne M. Bargman

**Affiliations:** The Home Peritoneal Dialysis Unit, University Health Network, University of Toronto, Toronto, Canada; Division of Nephrology, Toronto General Hospital, 200 Elizabeth Street, Toronto, ON M5G 2C4 Canada

**Keywords:** Encapsulating peritoneal sclerosis, Localized fibrosis, Peritoneal dialysis, Bowel obstruction

## Abstract

**Background:**

Encapsulating peritoneal sclerosis (EPS) is classically described as progressive sclerosis and cocooning of the entire peritoneum; however, there has been limited number of reported cases of localized fibrosis as a variant form.

**Case presentation:**

We describe two cases of acute bowel obstruction with isolated transition points in the setting of long-term peritoneal dialysis.

**Conclusion:**

We postulate that some of the cases of small bowel obstruction with an obvious transition point in long-term peritoneal dialysis patients may represent a unique and localized form of EPS. We aim to emphasize the presence of macroscopic variations in presentation of EPS.

## Background

Encapsulating peritoneal sclerosis (EPS) is a rare but devastating complication of long-term peritoneal dialysis (PD). It is characterized by progressive thickening and fibrosis of the peritoneal membrane, which eventually leads to encapsulation of the abdominal content by the abnormal fibrous tissue [1].

The incidence of EPS increases with time on PD, and presents most frequently after cessation of the therapy. Kawanishi et al. reported an incidence of 17.2 % after 15 years of PD therapy [2–5]. Similarly, the mortality rate from EPS increases with increased time on PD. For example, in the Pan-Thames EPS study, 42 and 100 % of patients succumbed at 3 and 15 years respectively [6]. Duration of exposure to PD therapy seems to be the principal risk factor for developing EPS, along with other currently identified factors including young age, genetic predisposition, and receipt of a kidney transplant.

There is an early inflammatory prodrome in many cases that can be missed. Manifestations include vague abdominal discomfort or anorexia, new-onset infusion pain, hemoperitoneum, and development of rapid transport status. Other clinical symptoms of EPS are directly related to disturbances in gastrointestinal transit, due to abnormal fibrous tissue formation around the bowel loops. The most common findings are abdominal pain, nausea, vomiting, anorexia, abdominal fullness, and eventually partial or complete small-bowel obstruction. Since the initial signs and symptoms of EPS are non-specific and subtle, a high index of suspicion is needed to establish an early diagnosis [7–9].

The classical macroscopic appearance of advanced EPS is total encapsulation of the bowel by a fibrotic membrane [10]. Laparotomy has usually revealed a thickened, brownish peritoneum with adhesions, and intestines that are partially or totally encapsulated in thick fibrous tissue. In advanced cases, a sclerotic layer has completely covered the intestines, giving the appearance of a cocoon [11, 12].

Localized involvement of parts of the bowel with fibrosis has been reported in the past, and it has been suggested that it could be more common than previously recognized [13, 14].

We report two cases of bowel obstruction with obvious transition point, appearing to be localized, which happened in patients after being on PD for many years. We postulate that localized fibrosis represents an atypical form of EPS.

## Case presentation

### Case 1

A 31-year-old woman was admitted with symptoms and signs of bowel obstruction. She had been diagnosed with systemic lupus erythematosus (SLE) about 14 years previously, and experienced multiple complications of SLE, including lupus nephritis, cerebritis and pleuritis. She had been on PD for 8 years. Other comorbidities included hypertension, hyperlipidemia and chronic anemia. She was on the waiting list for renal transplant at the time of presentation to the hospital. She had no history of peritonitis, and her PD treatment was uneventful. On initial 4-h peritoneal equilibrium test (PET), Dialysate/Plasma Creatinine (D/P Cr) was 0.71 (high-average transporter). Her lupus had otherwise been under control with no manifestations of active disease. There was no history of previous bowel surgeries or bowel obstruction.

She presented with a history of nausea, vomiting and abdominal pain, with report of not having had flatus or bowel movements for a few days.

**Fig. 1:**
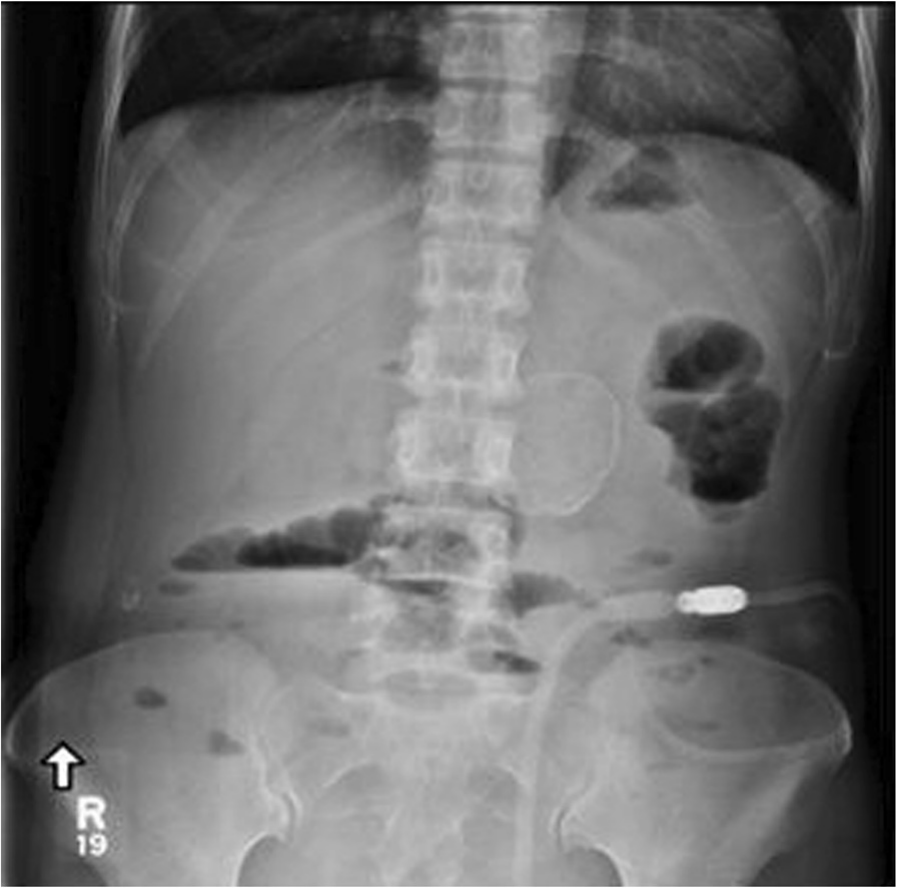
Plain abdominal radiography, upright view, showing several air-fluid levels

Plain radiography showed dilated small bowel loops, with several air-fluid levels on the erect view (Fig. 1).

**Fig. 2:**
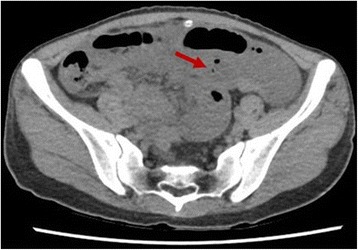
CT abdomen showing dilated bowel loops with transition point (arrow) in pelvis

To further investigate, computed tomography (CT) of the abdomen was obtained which showed dilated small bowel loops measuring up to 3.6 cm, with a transition point in the pelvis, where distal to this point the ileal loops were collapsed; the colon was relatively collapsed as well (Fig. 2).

Inflammatory markers included declining serum albumin level for the past few months with a nadir of 23 g/L, and erythrocyte sedimentation rate (ESR) of 38 mm/first hour.

The surgery service recommended non-operative management, with resting the bowels, and insertion of a nasogastric tube. The PD therapy was held in the setting of bowel obstruction; a hemodialysis line was inserted to initiate hemodialysis. The patient eventually recovered with this approach, and was tolerating oral intake upon discharge from the hospital. She elected to stay on hemodialysis after this presentation.

### Case 2

A 57-year-old female was admitted to the hospital with signs and symptoms of bowel obstruction.

Her past medical history included a diagnosis of anti-phospholipid antibody syndrome (APLAS) on anticoagulation, end-stage kidney disease, multiple previous arterial and venous thrombotic events, congestive heart failure, previous mitral valve replacement, atrial fibrillation, hepatitis C, and hypothyroidism. She had been started on PD in the form of continuous ambulatory PD (CAPD) 5 years prior to the current presentation. As with the first patient, there had been no peritonitis during her 5-year course of PD. There was no history of bowel surgery or bowel obstruction. On initial 4-h PET, D/P Cr was 0.78 (high-average transporter).

Upon presentation to the emergency department, she reported a 10-day history of nausea, vomiting, abdominal distension and pain, and absence of bowel movement.

**Fig. 3:**
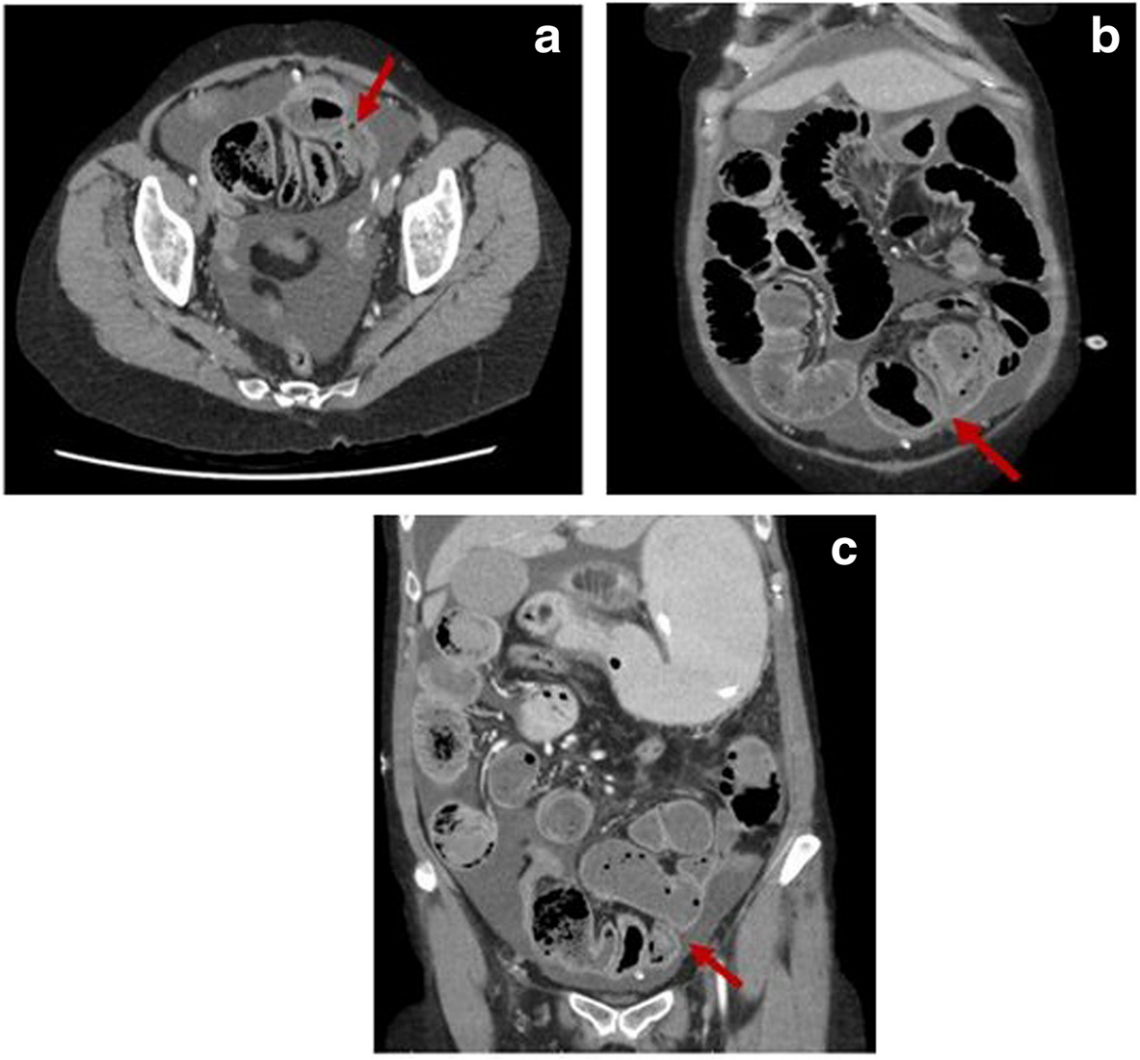
CT abdomen showing closed-loop small bowel obstruction, with multiple transition points (arrows: **a**, **b**, **c**) with abnormally displaced loops to the left lower pelvis (**b**, **c**)

To further investigate, a CT abdomen was performed which revealed fluid and gas distended loops of proximal small bowel, measuring up to 4.3 cm in diameter. This extended to the pelvis, where there was a focal cluster of distal loops displaced abnormally into the left lower pelvis with multiple transition points. The appearance was in keeping with a closed-loop distal small bowel obstruction (Fig. 3).

An exploratory laparotomy was performed during which a highly abnormal mass of small bowel loops was noted in the left lower quadrant, where the bowel loops could not be separated. Pathology report of the mass indicated a high degree of localized fibrous serosal adhesion. The abnormal mass of bowel loops was resected, with residual small bowel length of 170 cm. Similar to the previous case, serum albumin declined rapidly from a normal baseline over the preceding few months, with a nadir of 20 g/L. Other inflammatory markers were not obtained at this time. The PD catheter was removed during this operation, and the patient was permanently transitioned to hemodialysis.

## Discussion

We present two cases of bowel obstruction with transition point in the context of long term peritoneal dialysis. Patients with longstanding PD and concurrent autoimmune disease have previously identified as being particularly at risk for EPS [13]. EPS has classically been described as marked thickening and sclerosis of the entire peritoneal surface, a retraction of the root of the mesentery, and striking finding of encasement of the entire small bowel. There has been, however, limited number of case reports, describing “localized” EPS. Kirkman et al. reported a case of localized encapsulation of small bowel loops at the level of terminal ileum in a post-transplant patient previously on longstanding PD therapy [14].

Similarly Habib et al. reported 3 cases of “localized” EPS, where intra-operatively, an obvious sclerotic and thickened peritoneal membrane was observed, predominantly covering and constricting the terminal ileum [15].

By reporting these cases, although surgical exploration was not performed in our first case, we aim to emphasize the presence of macroscopic variations in presentation of EPS. We speculate that some of the cases of localized small bowel obstruction, with obvious transition points, in the context of prolonged PD therapy, may in fact represent non-classic forms of EPS. The approach to management of these patients should therefore be similar to those proposed for the more generalized form of encapsulation. A conservative approach with resting the bowels should be tried, and in cases where the index of suspicion for EPS is significant, we suggest termination of PD and switching to a different dialysis modality. In case of persistent bowel obstruction despite conservative management, a surgical approach is suitable, as described in our second case, in addition to termination of PD. Further case studies are needed to investigate the use of pharmacologic agents such as tamoxifen or corticosteroids.

## Conclusion

It is suggested that clinicians have a high index of suspicion for EPS in patients on long-term PD therapy who present with acute bowel obstruction with an isolated transition point.

## Consent

The patients provided full informed consent for gathering the data and publishing the case.

## Abbreviations

ESKD: End stage kidney disease
PD: Peritoneal dialysis
CAPD: Continuous ambulatory peritoneal dialysis
SLE: Systemic lupus erythromatosis
EPS: Encapsulating peritoneal sclerosis
